# Supplementary material for: Blood Oxygenation Level–Dependent Cerebrovascular Reactivity–Derived Steal Phenomenon May Indicate Tissue Reperfusion Failure After Successful Endovascular Thrombectomy
Source: Transl Stroke Res. 2023 Oct 25;16(2):207–16. doi: 10.1007/s12975-023-01203-y (PMC11976757; doi:10.1007/s12975-023-01203-y)
Supplement: Supplementary file 1 — Supplementary file1 (DOCX 220 KB) [file 12975_2023_1203_MOESM1_ESM.docx]

# 1 SUPPLEMENTAL MATERIALS AND METHODS

2
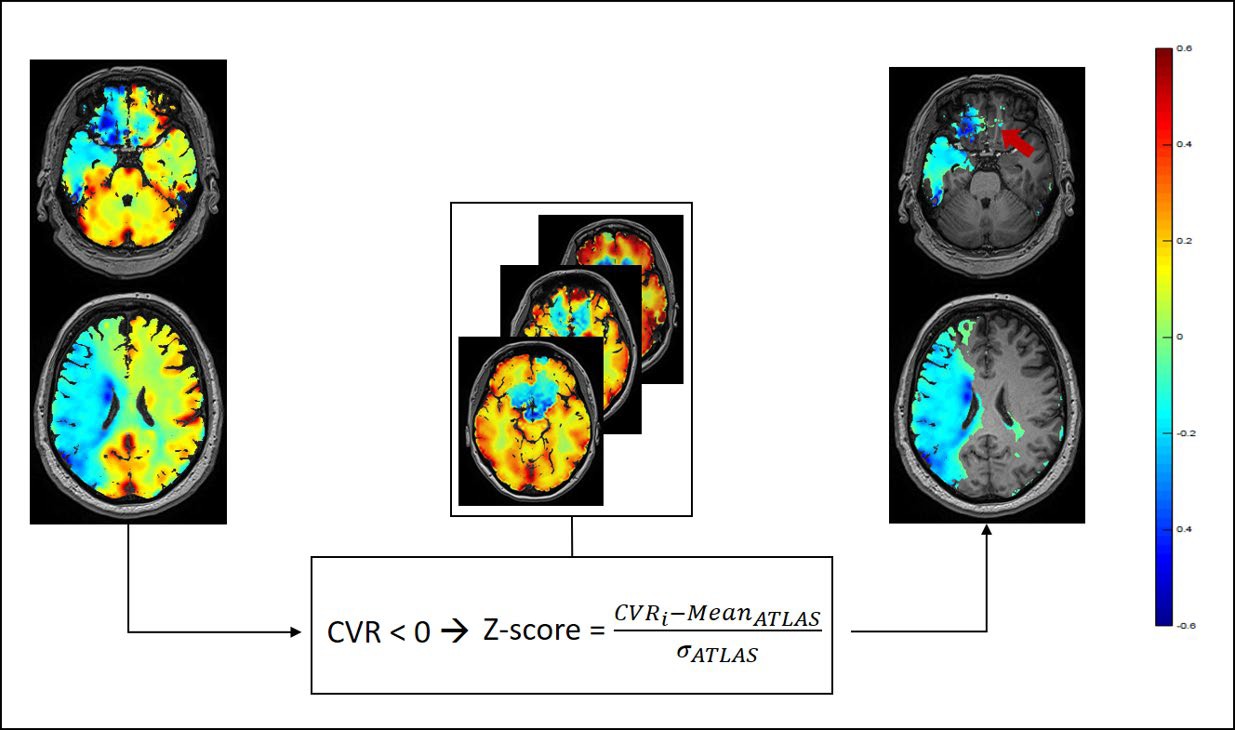


# Supplementary Figure 1. BOLD-CVR associated steal phenomenon mask generation.

## For each patient the relevant voxels showing negative response in the blood oxygen level

1. dependent cerebrovascular reactivity (BOLD-CVR) map were selected. First, we considered
2. all voxels with < 0% BOLD signal change/mmHg CO2. Then, using our healthy atlas as
3. reference, a Z-score map of the CVR map was generated and the voxels with Z-score < 2
4. were excluded. In this way, only those negative voxels that differed significantly from the
5. healthy cohort (e.g., voxels with artefact-related negative BOLD signal in the frontobasal
6. brain region, red arrow) were considered.

11


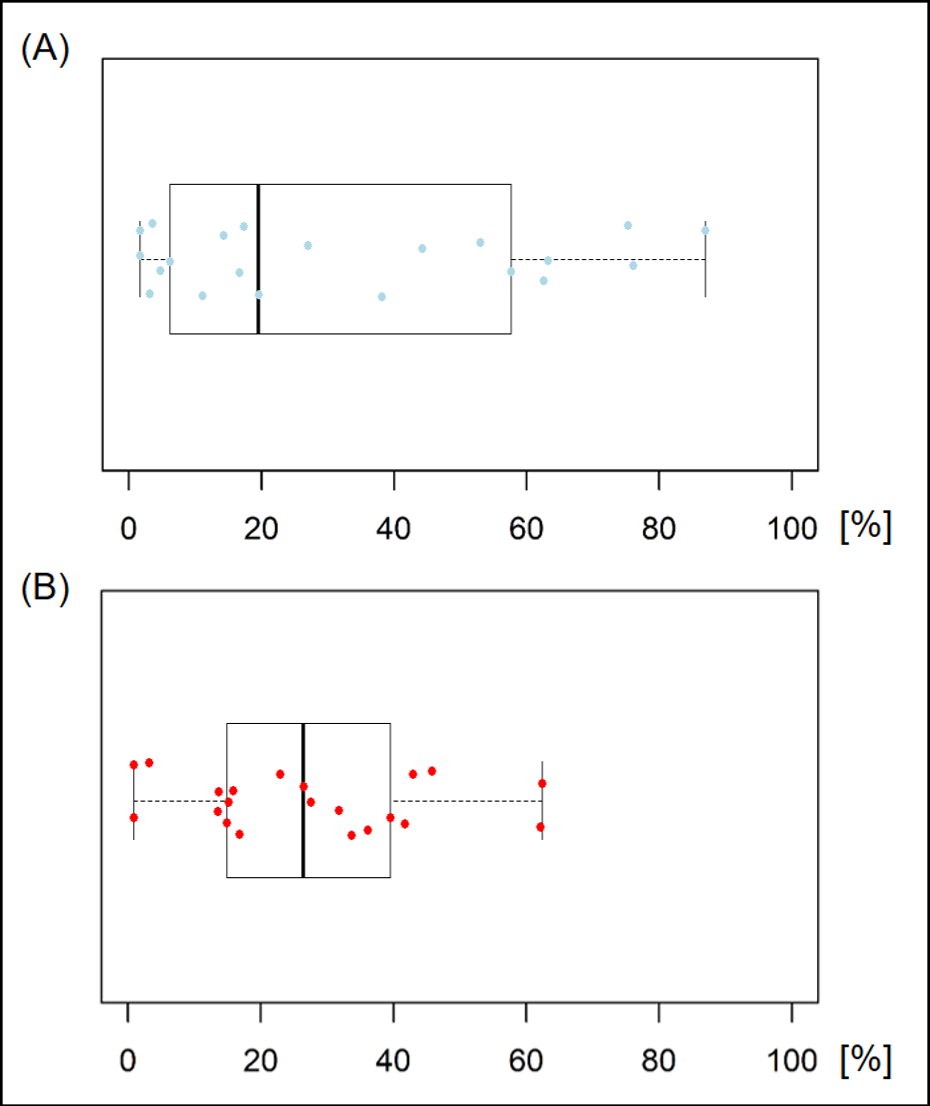
12

# Supplementary Figure 2. Spatial correlation between BOLD-CVR associated steal

1. **phenomenon and DWI infarct lesion.**

## BOLD-CVR indicates blood oxygen level dependent cerebrovascular reactivity. **A**, a boxplot

1. of the percentage of DWI infarct lesion included in the BOLD-CVR associated steal
2. phenomenon; **B**, a boxplot of the percentage of BOLD-CVR associated steal phenomenon
3. included in the DWI infarct lesion.

19

TA: 49 sec Coil Selection: Manual Voxel Size: 1.5×1.5×4.5 mm³ Acc:: 4 Rel. SNR: 1.00

**\\USER\test\test\CRPPstroke20191228_32ch_New_Patients6-dwicvrt1\ep2d_diff_mddw_12_p2s2 ***

Properties

| Start measurement without further Off preparation  Wait for User to Start Off  Start measurements Single Measurement  Prio Recon Off  Auto Open Inline Display Off  Auto Close Inline Display Off  Load Images to MR View&GO On  Auto Store Images On Load Images to Stamp Segments Off Load Images to Graphic Segments Off Graphic segment Default  Inline Movie Off |
| --- |

Routine

| Slice Group | 1 |
| --- | --- |
| Slices | 32 |
| Distance Factor | 12 % |
| Position | R1.0 P15.5 H21.2 mm |
| Orientation | T > C-12.5 > S4.7 |
| Phase Encoding Dir. | A >> P |
| Phase Oversampling | 0 % |
| FoV Read | 240 mm |
| FoV Phase | 100.0 % |
| Slice Thickness | 4.5 mm |
| TR | 2500.0 ms |
| TE | 75.00 ms |
| Concatenations | 1 |
| AutoAlign | Head > Brain |
| Coil Elements | HEA;HEP |

Contrast - Common

| TR 2500.0 ms  TE 75.00 ms  MTC Off  Magn. Preparation None  Fat-Water Contrast Fat Saturation  Fat Saturation Strong  Reconstruction Magnitude |
| --- |

Contrast - Dynamic

| Dynamic Mode Standard  Multiple Series Off  Delay in TR 0.00 ms |
| --- |

Resolution - Common

| FoV Read 240 mm  FoV Phase 100.0 %  Slice Thickness 4.5 mm  Base Resolution 160  Phase Resolution 100 %  Interpolation Off |
| --- |

Resolution - Acceleration

| Acceleration mode SMS  Reference Scans EPI/Separate  Acceleration Factor PE 2  Reference Lines PE 38  SMS Factor 2  Phase Partial Fourier 6/8 |
| --- |

Resolution - Filter

| Raw Filter Off  Elliptical Filter Off  Distortion Correction Off  Normalize Prescan |
| --- |

Geometry - Common

| Slice Group | 1 |
| --- | --- |
| Slices | 32 |
| Distance Factor | 12 % |
| Position | R1.0 P15.5 H21.2 mm |
| Orientation | T > C-12.5 > S4.7 |
| Phase Encoding Dir. | A >> P |
| Phase Oversampling | 0 % |
| FoV Read | 240 mm |
| FoV Phase | 100.0 % |
| Slice Thickness | 4.5 mm |
| TR | 2500.0 ms |
| Multi-Slice Mode | Interleaved |
| Series | Interleaved |
| Concatenations | 1 |

Geometry - AutoAlign

| Slice Group | 1 |
| --- | --- |
| Position Orientation  Phase Encoding Dir. | R1.0 P15.5 H21.2 mm T > C-12.5 > S4.7  A >> P |
| AutoAlign | Head > Brain |
| Initial Position | L0.4 A3.2 F0.3 |
| L | 0.4 mm |
| A | 3.2 mm |
| F | 0.3 mm |
| Initial Orientation | T > C |
| T > C | -4.40 |
| > S | 0.10 |
| Initial Rotation | 1.70 deg |

Geometry - Navigator Geometry - Saturation

| Special Saturation None |
| --- |

Geometry - Tim Planning Suite

| Set-n-Go Protocol Off  Table Position 0 mm  Table Position H  Inline Composing Off |
| --- |

System - Miscellaneous

| Coil Selection Manual  MSMA S - C - T  Sagittal R >> L  Coronal A >> P  Transversal F >> H  Coil Combination Adaptive Combine  Matrix Optimization Off |
| --- |

System - Adjustments

| Adjustment Strategy Standard  B0 Shim Advanced  B1 Shim TrueForm |
| --- |

System - Adjustments

| Adjustment Tolerance Auto  Adjust with Body Coil Off  Confirm Frequency Never  Assume Silicone Off |
| --- |

System - Adjust Volume

| Position R1.0 P15.5 H21.2 mm  Orientation T > C-12.5 > S4.7  Rotation 3.40 deg  A >> P 240 mm  R >> L 240 mm  F >> H 161 mm  Reset Off |
| --- |

System - Tx/Rx

| Frequency 1H 123.256368 MHz  ? Ref. Amplitude 1H 0.000 V  Reset Off  Correction Factor 1.00  Image Scaling 1.000 |
| --- |

Physio - Signal

| 1st Signal/Mode None  TR 2500.0 ms  Concatenations 1 |
| --- |

Physio - PACE

| Resp. Control Off  Concatenations 1 |
| --- |

Diff

| Diffusion Mode MDDW  Diff. Directions 12  Diffusion Scheme Monopolar  Diff. Weightings 2  b-value 1 0 s/mm²  b-value 2 1000 s/mm²  Averages 1 1  Averages 2 1  Dynamic Field Correction On  Invert Gray Scale Off  Diff. Weighted Images On  Trace Weighted Images On  Tensor Off  FA Maps Off  ADC Maps On  Exponential ADC Maps Off  ADC Noise Threshold 40  Noise Masking Off  Calculated Image Off |
| --- |

Sequence - Part 1

Sequence - Part 2

| Introduction Off  Phase Correction Internal |
| --- |

| Sequence Name epse  Excitation Standard  RF Pulse Type Normal  Gradient Mode Fast  Bandwidth 1202 Hz/Px  Echo Spacing 0.94 ms  Free Echo Spacing Off  Optimization None  EPI Factor 160 |
| --- |

TA: 6:46 min Coil Selection: Auto Voxel Size: 3.0×3.0×3.0 mm³ Acc:: 2 Rel. SNR: 1.00

**\\USER\test\test\CRPPstroke20191228_32ch_New_Patients6-dwicvrt1\CO2_bold ***

Properties

| Start measurement without further On preparation  Wait for User to Start Off  Start measurements Single Measurement  Prio Recon Off  Auto Open Inline Display Off  Auto Close Inline Display Off  Load Images to MR View&GO On  Auto Store Images On Load Images to Stamp Segments Off Load Images to Graphic Segments Off Graphic segment Default  Inline Movie Off |
| --- |

Routine

| Slice Group | 1 |
| --- | --- |
| Slices | 35 |
| Distance Factor | 10 % |
| Position | R0.8 P5.9 H10.6 mm |
| Orientation | T > C-16.5 > S0.6 |
| Phase Encoding Dir. | A >> P |
| Phase Oversampling | 0 % |
| FoV Read | 192 mm |
| FoV Phase | 100.0 % |
| Slice Thickness | 3.0 mm |
| TR | 2000.0 ms |
| TE | 30.00 ms |
| Averages | 1 |
| Concatenations | 1 |
| AutoAlign | Head > Brain |
| Coil Elements | HEA;HEP |

Contrast - Common

| TR 2000.0 ms  TE 30.00 ms  MTC Off  Flip Angle 85 deg  Fat-Water Contrast Fat Saturation  Reconstruction Magnitude |
| --- |

Contrast - Dynamic

| Dynamic Mode Standard  Measurements 200  Delay in TR 0.00 ms |
| --- |

Resolution - Common

| FoV Read 192 mm  FoV Phase 100.0 %  Slice Thickness 3.0 mm  Base Resolution 64  Phase Resolution 100 %  Interpolation Off |
| --- |

Resolution - Acceleration

| Acceleration mode GRAPPA  Reference Scans EPI/Separate  Acceleration Factor PE 2  Reference Lines PE 32  Phase Partial Fourier Off |
| --- |

Resolution - Filter

| Raw Filter Off  Elliptical Filter On  Hamming Off  Distortion Correction Off  Normalize Prescan |
| --- |

Geometry - Common

| Slice Group | 1 |
| --- | --- |
| Slices | 35 |
| Distance Factor | 10 % |
| Position | R0.8 P5.9 H10.6 mm |
| Orientation | T > C-16.5 > S0.6 |
| Phase Encoding Dir. | A >> P |
| Phase Oversampling | 0 % |
| FoV Read | 192 mm |
| FoV Phase | 100.0 % |
| Slice Thickness | 3.0 mm |
| TR | 2000.0 ms |
| Multi-Slice Mode | Interleaved |
| Series | Interleaved |
| Concatenations | 1 |

Geometry - AutoAlign

| Slice Group | 1 |
| --- | --- |
| Position Orientation  Phase Encoding Dir. | R0.8 P5.9 H10.6 mm T > C-16.5 > S0.6  A >> P |
| AutoAlign | Head > Brain |
| Initial Position | R0.8 P5.9 H10.6 |
| R | 0.8 mm |
| P | 5.9 mm |
| H | 10.6 mm |
| Initial Orientation | T > C |
| T > C | -16.50 |
| > S | 0.60 |
| Initial Rotation | -1.12 deg |

Geometry - Saturation

| Special Saturation None |
| --- |

Geometry - Tim Planning Suite

| Set-n-Go Protocol Off  Table Position 0 mm  Table Position H  Inline Composing Off |
| --- |

System - Miscellaneous

| Coil Selection Auto Coil Select  MSMA S - C - T  Sagittal R >> L  Coronal A >> P  Transversal F >> H  Coil Combination Adaptive Combine  Matrix Optimization Off |
| --- |

System - Adjustments

| Adjustment Strategy Standard  B0 Shim Standard  B1 Shim TrueForm  Adjustment Tolerance Auto |
| --- |

System - Adjustments

| Adjust with Body Coil Off  Confirm Frequency Never  Assume Silicone Off |
| --- |

System - Adjust Volume

| Position R0.8 P5.9 H10.6 mm  Orientation T > C-16.5 > S0.6  Rotation -1.12 deg  A >> P 192 mm  R >> L 192 mm  F >> H 116 mm  Reset Off |
| --- |

System - Tx/Rx

| Frequency 1H 123.256368 MHz  ? Ref. Amplitude 1H 0.000 V  Reset Off  Correction Factor 1.00  Image Scaling 1.000 |
| --- |

Physio - Signal

| 1st Signal/Mode None  TR 2000.0 ms  Concatenations 1 |
| --- |

BOLD

| GLM Statistics Off  Ignore Meas. at Start 0  Ignore After Transition 0  Model Transition States On  Temp. Highpass Filter On  Threshold 2.50  Paradigm Size 66  Meas[1] Active  Meas[2] Active  Meas[3] Active  Meas[4] Active  Meas[5] Active  Meas[6] Active  Meas[7] Active  Meas[8] Active  Meas[9] Active  Meas[10] Active  Meas[11] Active  Meas[12] Active  Meas[13] Active  Meas[14] Active  Meas[15] Active  Meas[16] Active  Meas[17] Active  Meas[18] Active  Meas[19] Active  Meas[20] Active  Meas[21] Active  Meas[22] Active  Meas[23] Ignore  Meas[24] Ignore  Meas[25] Ignore  Meas[26] Ignore  Meas[27] Ignore  Meas[28] Ignore  Meas[29] Ignore  Meas[30] Ignore  Meas[31] Ignore |
| --- |

BOLD

| Meas[32] Ignore  Meas[33] Ignore  Meas[34] Ignore  Meas[35] Ignore  Meas[36] Ignore  Meas[37] Ignore  Meas[38] Ignore  Meas[39] Ignore  Meas[40] Ignore  Meas[41] Ignore  Meas[42] Ignore  Meas[43] Ignore  Meas[44] Ignore  Meas[45] Active  Meas[46] Active  Meas[47] Active  Meas[48] Active  Meas[49] Active  Meas[50] Active  Meas[51] Active  Meas[52] Active  Meas[53] Active  Meas[54] Active  Meas[55] Active  Meas[56] Active  Meas[57] Active  Meas[58] Active  Meas[59] Active  Meas[60] Active  Meas[61] Active  Meas[62] Active  Meas[63] Active  Meas[64] Active  Meas[65] Active  Meas[66] Active  Motion Correction On  Spatial Filter On  Filter Width 4.0 mm  Measurements 200  Delay in TR 0.00 ms |
| --- |

Sequence - Part 1

| Sequence Name epfid  Excitation Standard  RF Pulse Type Normal  Gradient Mode Fast*  Bandwidth 2368 Hz/Px  Echo Spacing 0.53 ms  Free Echo Spacing Off  EPI Factor 64 |
| --- |

Sequence - Part 2

| Introduction Off |
| --- |

TA: 8:14 min Coil Selection: Auto Voxel Size: 0.8×0.8×1.0 mm³ Acc:: 2 Rel. SNR: 1.00

**\\USER\test\test\CRPPstroke20191228_32ch_New_Patients6-dwicvrt1\t1mprage_tra_HighRes_Nch_ 32ch ***

Properties

| Start measurement without further On preparation  Wait for User to Start Off  Start measurements Single Measurement  Prio Recon Off  Auto Open Inline Display Off  Auto Close Inline Display Off  Load Images to MR View&GO On  Auto Store Images On Load Images to Stamp Segments Off Load Images to Graphic Segments Off Graphic segment Default  Inline Movie Off |
| --- |

Routine

| Slab Group | 1 | |
| --- | --- | --- |
| Slabs | 1 |  |
| Distance Factor | 50 % |  |
| Position | L0.0 P1.6 H13.6 | mm |
| Orientation | Transversal |  |
| Phase Encoding Dir. | R >> L |  |
| Slices per Slab | 176 | |
| Phase Oversampling | 10 % | |
| Slice Oversampling | 27.3 % | |
| FoV Read | 230 mm | |
| FoV Phase | 100.0 % | |
| Slice Thickness | 1.0 mm | |
| TR | 2200.0 ms | |
| TE | 5.17 ms | |
| Averages | 1 | |
| Concatenations | 1 | |
| AutoAlign | Head > Brain | |
| Coil Elements | HEA;HEP | |

Contrast - Common

| TR 2200.0 ms  TE 5.17 ms  Magn. Preparation Non-sel. IR  TI 900 ms  Flip Angle 8 deg  Fat-Water Contrast Standard  Dark Blood Off  Reconstruction Magnitude |
| --- |

Contrast - Dynamic

| Dynamic Mode Standard  Measurements 1  Multiple Series Each Measurement  Reordering Linear Rot. |
| --- |

Resolution - Common

| FoV Read 230 mm  FoV Phase 100.0 %  Slice Thickness 1.0 mm  Base Resolution 288  Phase Resolution 100 %  Slice Resolution 100 %  Interpolation Off |
| --- |

Resolution - Acceleration

| Acceleration mode GRAPPA  Reference Scans Integrated  Acceleration Factor PE 2  Reference Lines PE 24  Acceleration Factor 3D 1  Phase Partial Fourier Off  Slice Partial Fourier Off  Asymmetric Echo Allowed  Elliptical Scanning Off |
| --- |

Resolution - Filter

| Raw Filter Off  Elliptical Filter Off  Distortion Correction 3D  Normalize Prescan  Image Filter On |
| --- |

Geometry - Common

| Slab Group | 1 |
| --- | --- |
| Slabs | 1 |
| Distance Factor | 50 % |
| Position | L0.0 P1.6 H13.6 mm |
| Orientation | Transversal |
| Phase Encoding Dir. | R >> L |
| Slices per Slab | 176 |
| Phase Oversampling | 10 % |
| Slice Oversampling | 27.3 % |
| FoV Read | 230 mm |
| FoV Phase | 100.0 % |
| Slice Thickness | 1.0 mm |
| TR | 2200.0 ms |
| Multi-Slice Mode | Single Shot |
| Series | Ascending |
| Concatenations | 1 |

Geometry - AutoAlign

| Slab Group | 1 |
| --- | --- |
| Position Orientation  Phase Encoding Dir. | L0.0 P1.6 H13.6 mm  Transversal R >> L |
| AutoAlign | Head > Brain |
| Initial Position | L0.0 P1.6 H13.6 |
| R | 0.0 mm |
| P | 1.6 mm |
| H | 13.6 mm |
| Initial Orientation | Transversal |
| Initial Rotation | 91.66 deg |

Geometry - Navigator Geometry - Tim Planning Suite

| Set-n-Go Protocol Off  Table Position 0 mm  Table Position H  Inline Composing Off |
| --- |

System - Miscellaneous

| Coil Selection Auto Coil Select  MSMA S - C - T |
| --- |

System - Miscellaneous

| Sagittal R >> L  Coronal A >> P  Transversal F >> H  Coil Combination Adaptive Combine  Matrix Optimization Off |
| --- |

System - Adjustments

| Adjustment Strategy Standard  B0 Shim Tune up  B1 Shim TrueForm  Adjustment Tolerance Auto  Adjust with Body Coil Off  Confirm Frequency Never  Assume Silicone Off |
| --- |

System - Adjust Volume

| Position Isocenter  Orientation Transversal  Rotation 0.00 deg  A >> P 263 mm  R >> L 350 mm  F >> H 350 mm  Reset Off |
| --- |

System - Tx/Rx

| Frequency 1H 123.256368 MHz  ? Ref. Amplitude 1H 0.000 V  Reset Off  Correction Factor 1.00  Image Scaling 1.000 |
| --- |

Physio - Signal

| 1st Signal/Mode None  TR 2200.0 ms  Concatenations 1 |
| --- |

Physio - Cardiac

| Fat-Water Contrast Standard  Magn. Preparation Non-sel. IR  TI 900 ms  Dark Blood Off  FoV Read 230 mm  FoV Phase 100.0 %  Phase Resolution 100 %  Dynamic Mode Standard |
| --- |

Physio - PACE

| Resp. Control Off  Concatenations 1 |
| --- |

Inline - Subtraction

| Subtract Off  Measurements 1  StdDev Off  Save Original Images On |
| --- |

Inline - Cardiac

Inline - MIP

| MIP Sag Off  MIP Cor Off  MIP Tra Off  MIP Time Off  Radial MIP Off  Save Original Images On  MPR Sag Off  MPR Cor Off  MPR Tra Off |
| --- |

Inline - Composing

| Inline Composing Off |
| --- |

Sequence - Part 1

| Sequence Name tfl_r  Dimension 3D  Excitation Slab-sel.  RF Pulse Type Fast  Gradient Mode Normal  Flow Compensation On  Reordering Linear Rot.  Bandwidth 250 Hz/Px  Echo Spacing 10.18 ms  Asymmetric Echo Allowed  Turbo Factor 317 |
| --- |

Sequence - Part 2

| Introduction On  RF Spoiling On  Incr. Gradient Spoiling On |
| --- |

Sequence - Assistant

| SAR Assistant Off |
| --- |

| Magn. Preparation Non-sel. IR  Save Original Images On  TE 5.17 ms  TR 2200.0 ms |
| --- |
